# Retraction Note: Fibulin-3 knockdown inhibits cervical cancer cell growth and metastasis in vitro and in vivo

**DOI:** 10.1038/s41598-023-38389-y

**Published:** 2023-07-10

**Authors:** Juan Li, Chen Qi, Xia Liu, Changzhong Li, Jie Chen, Min Shi

**Affiliations:** 1 Department of Obstetrics and Gynecology, Shandong Provincial Hospital Affiliated to Shandong University, Jinan, 250021 China; 2 Department of Obstetrics and Gynecology, Shan Xian Maternal and Child Care and Family Planning Service Center, Shan Xian, 274300 China; 3 Department of Maternal and Child Health, School of Public Health, Shandong University, Jinan, 250012 China

Retraction of: *Scientific Reports* 10.1038/s41598-018-28906-9, published online 13 July 2018

The Editors have retracted this Article.

After publication of this Article it was brought to the Editors’ attention that some of the images appear to overlap with those in other published articles partially from the same author group, where the data is partially attributed to different samples. Specifically:

- In Fig 2C the “SiHa-23” group appears to partially overlap with Fig 2C “A19” group in^1^;
- In Fig 4D the “Fibulin-3 cDNA, HeLa-25” group appears to be rotated and partially overlaps with Fig 3C “U-2OS” group in^2^;
- In Fig 8B the “N-cadherin” group (left panel, right side of image) appears to partially overlap with Fig 8C “Vimentin” group (left panel, left side of image).

The Editors reached out to the Authors to request raw data. The Authors provided the data which did not address the concerns. The Editors therefore no longer have confidence in the results presented in this Article.

Juan Li, Chen Qi, Xia Liu, Changzhong Li, and Jie Chen did not respond to the correspondence about this retraction. Min Shi did not state explicitly whether they agree to or disagree with this retraction.
